# A prognostic value of CD45RA^+^, CD45RO^+^, CCL20^+^ and CCR6^+^ expressing cells as ‘immunoscore’ to predict cervical cancer induced by HPV

**DOI:** 10.1038/s41598-021-88248-x

**Published:** 2021-04-22

**Authors:** Ana Teresa G. Fernandes, Maria Odete O. Carvalho, Elyzabeth Avvad-Portari, Natália P. Rocha, Fabio Russomano, Eric Henrique Roma, Maria da Gloria Bonecini-Almeida

**Affiliations:** 1 Laboratory of Immunology and Immunogenetics in Infectious Diseases at Evandro Chagas National Institute of Infectious Diseases, Oswaldo Cruz Foundation, Avenida Brasil 4365, Rio de Janeiro, RJ 21040-900 Brazil; 2 Department of Pathologic Anatomy at Fernandes Figueira Woman, Child and Adolescent’s Health National Institute, Oswaldo Cruz Foundation, Rio de Janeiro, Brazil; 3 Department of Gynecology at Fernandes Figueira Woman, Child and Adolescent’s Health National Institute, Oswaldo Cruz Foundation, Rio de Janeiro, Brazil

**Keywords:** Cancer, Immunology

## Abstract

The interplay between cervical cancer (CC) and immune cells, mainly intratumoral lymphocytes, has a pivotal role in carcinogenesis. In this context, we evaluated the distribution of CD45RA^+^ and CD45RO^+^ cells as well as CCR6^+^ and CCL20^+^ cells in intraepithelial (IE) and marginal stroma (MS) areas from cervical intraepithelial neoplasia (CIN) I–III, and CC as ‘immunoscore’ for HPV-induced CC outcome. We observed increased CD45RA^+^ and CD45RO^+^ cells distribution in IE and MS areas in the CC group compared to CIN groups and healthy volunteers. Interestingly, there is a remarkable reduction of CCL20^+^ expressing cells distribution according to lesion severity. The CC group had a significant decrease in CCL20^+^ and CCR6^+^-expressing cells distribution in both IE and MS areas compared to all groups. Using the ‘immunoscore’ model, we observed an increased number of women presenting high CD45RA^+^/CD45RO^+^ and low CCL20^+^/CCR6^+^ ‘immunoscore’ in the CC group. Our results suggested a pattern in cervical inflammatory process with increasing CD45RA^+^/CD45RO^+^, and decreasing CCL20^+^/CCR6^+^ expression in accordance with CIN severity. Taken together, these markers could be evaluated as ‘immunoscore’ predictors to CC response. A more comprehensive analysis of longitudinal studies should be conducted to associate CD45RA^+^/CD45RO^+^ and CCL20^+^/CCR6^+^ ‘immunoscore’ to CC progression and validate its value as a prognosis method.

## Introduction

Despite advances in prevention and early detection, cervical cancer (CC) is the fourth most common type of cancer in the female population worldwide^1^ and is associated with the human papillomavirus (HPV) infection in 99.7% of CC cases^2^. Increasing evidence demonstrates that the evolution of cancer is strongly dependent on the complex tumor microenvironment (TME) that comprises fibroblasts, endothelial cells, blood vessels, lymph vessels, and immune cells. Adaptive immune cell infiltration was shown to have a prognostic value superior to the classic tumor invasion criteria, including grade, stage, and metastatic status^3,4^.

The immune response against HPV antigens may control the infection and promote lesion regression, probably through a CD4^+^ Th1 response against the E2, E6, and E7 proteins^5,6^. In our previous study, higher amounts of intraepithelial CD4^+^, CD8^+^ T cells and macrophages were observed in women with CC precursor lesions when compared to healthy volunteers, indicating that cellular immune response has an important role in HPV-associated cervical intraepithelial neoplasia (CIN)^7^. However, the lymphocyte populations in the cervical mucosal tissues, especially cervical intraepithelial lymphocytes, have been poorly studied. T cell activation requires CD45 family-signaling transduction, a transmembrane tyrosine phosphatase expressed in all nucleated hematopoietic cells^8,9^. In T cells, CD45 molecule indicates different stages of maturation and activation, and CD45RA and CD45RO isoforms are expressed in naïve and activated T cells, respectively. Few reports have described the role of naïve (CD45RA^+^) and memory (CD45RO^+^) T cells in HPV-associated cancers. HrHPVs interfere with immune mediators in order to suppress the recruitment of immune cells and antiviral activities of infected cells. E6 and E7 from hrHPVs may downregulate the CCL20 chemokine production in keratinocytes, by inhibiting its transcription, resulting in suppression of CCR6^+^ inflammatory cell migration and immune system escape^10^.

Currently, most prognostic models for CC associate clinical markers with an increased risk for disease recurrence or death^11–13^. Some studies have evaluated how tumor-infiltrating immune cells (TICs) interact with cancer cells, shape the TME and affect clinical outcomes^14^. In CC, inflammation induced by HPV leads to a relatively complex TIC composition that plays an important role in tumorigenesis^15^. To determine the prognostic value of TICs, their fractions have been analyzed in CC and it can accurately estimate the immune composition in a tumor fragment^16^, performing a comprehensive exploration of the predictive value of TIC subpopulations. The most of the enriched cell infiltrates are CD8^+^ T cells and macrophages, especially in stromal area in CC. The infiltration pattern in CC was significantly different from normal control tissue, whose infiltration is composed of memory CD4^+^ T cells^17^. The distinct infiltration pattern indicated that the variation in TICs is an intrinsic feature that could be used to characterize individual differences in cervical cancer and could have important clinical meaning^18^. Besides, studies have identified immune-related genes, including those enriched for T cell activation, membrane-related, carbohydrate binding, and cytokine-cytokine receptor interaction, which could be used as promising biomarkers for the prognostic of CC^19^.

Recently, new methods based on the density of lymphocyte populations have been established to predict the outcome of colon cancer^20–22^. This method, known as ‘immunoscore’ incorporated the number, type, and distribution of immune cells in cancer tissue. In cervical lesions, it may be applied in the CIN-II diagnosis, through the identification of Ki67 and p16 biomarkers^23^ or in study of tumor-induced immune suppression and escape, mediated by the programmed cell death receptor 1 (PD-1) and its ligand (PD-L1)^24^.

Understanding the role of the cervical immune microenvironment in precursor lesions is crucial for impact on the progression to CC. This effective predictive model using the ‘immunoscore’ method compared to adopted staging systems, as tumor/node/metastasis (TNM), is more practicable, and should be introduced to support clinicians to identify patients at higher risk of poor prognosis. Our goals were to first describe the distribution and density of intraepithelial and stromal marginal CD45RA^+^, CD45RO^+^, CCR6^+^ and CCL20^+^ expressing cells to establish a prognostic ‘immunoscore’ to CC progression.

## Results

### Characteristics of the study population

Clinical and environmental data were summarized in Table 1. Patients (n = 67) were sub-grouped in accordance with their clinical status in CIN I (n = 17), CIN II (n = 16), CIN III (n = 19) and CC (n = 15). Fourteen volunteers were included as healthy control (HC) group. There were no differences in age among the CIN subgroups. However, women with CC were older compared to HC (*p* < 0.01), CIN I (*p* < 0.01), CIN II (*p* < 0.01) and CIN III (*p* < 0.001). CIN I subgroup showed a higher percentage of tobacco users when compared to CIN II (*p* < 0.01) and CIN III (*p* < 0.001) groups. CIN I subgroup showed higher percentage of women smokers than CIN II (*p* < 0.01) and CIN III (*p* < 0.001) subgroups. No statistical difference was observed related to ethnicity, alcohol consumption, age at first sexual intercourse, number of partners, and number of abortions. *P* value was calculated using Anova test and Dunn's Multiple Comparison Post Test for quantitative variables, and χ^2^ Test for qualitative variables.

**Table 1:** Clinical and environmental data in cervical HPV-associated lesion and cancer.

	Control (n = 14)	CIN			CC** (n = 15)
		CIN I** (n = 17)	CIN II (n = 16)	CIN III (n = 19)	
Age years (mean ± SD)*	31.9 ± 5.3^a^	33.1 ± 10.4^b^	31.90 ± 7.3^c^	32.2 ± 8.2^d^	47.9 ± 8.9
Ethinicity n (%)**					
White	7 (50.0)	7 (41.2)	10 (62.5)	8 (47.1)	5 (33.3)
Afro-Brazilian	7 (50.0)	10 (58.8)	6 (37.5)	11 (52.9)	10 (66.7)
Alcohol consumption n (%)**					
Yes	4 (28.5)	7 (41.2)	6 (37.5)	9 (47.4)	5 (33.3)
No	10 (71.5)	10 (58.8)	10 (62.5)	10 (52.6)	10 (66.7)
Tobacoo use n (%)**					
Yes	4 (28.5)	10 (58.8)	3 (18.7)^e^	2 (10.5)^f^	5 (33.3)
No	10 (71.5)	7 (41.2)	13 (81.3)	17 (89.5)	10 (66.7)
Age at first sexual intercourse (average ± SD)*	18.4 ± 2.4	18.1 ± 3.2	16.9 ± 2.7	16.7 ± 2.7	17.2 ± 1.3
Number of partners (average ± SD)*	4.0 ± 2.9	3.4 ± 1.7	6.7 ± 8.6	5.7 ± 4.7	4.0 ± 1.2
Number of abortions (average ± SD)*	1 ± 0.9	0.7 ± 0.9	0.3 ± 0.8	0.3 ± 0.4	0.7 ± 0.8
Positive HPV DNA n (%)	0 (0)	15 (88.2)	14 (87.5)	18 (94.7)	15 (100)

*One way Anova test and Dunn's multiple comparison post test; **χ^2^ test.

**CIN cervical intraepithelia neoplasia, *CC* cervical cancer.

^a^*p* < 0.01 control versus CC, ^b^*p* < 0.01 CIN I versus CC; ^c^*p* < 0.01 CIN II versus CC; ^d^*p* < 0.001, CIN III versus CC; ^e^*p* < 0.01 CIN II versus CIN I; ^f^*p* < 0.001 CIN III versus CIN I.

Among CIN subgroups, 47 (90.3%) women were positive for HPV-DNA. In the remaining 5 CIN patients (3 CIN I and 2 CIN II), due to the poor quality of the DNA tissue extracted, it was not possible to detect HPV-DNA. In CC group, HPV-DNA was identified in all patients, and was absent in all HC volunteers (Table 1).

### Distribution of CD45RA^+^ and CD45RO^+^ cells increase according to cervical lesions severity

**Figure 1:**
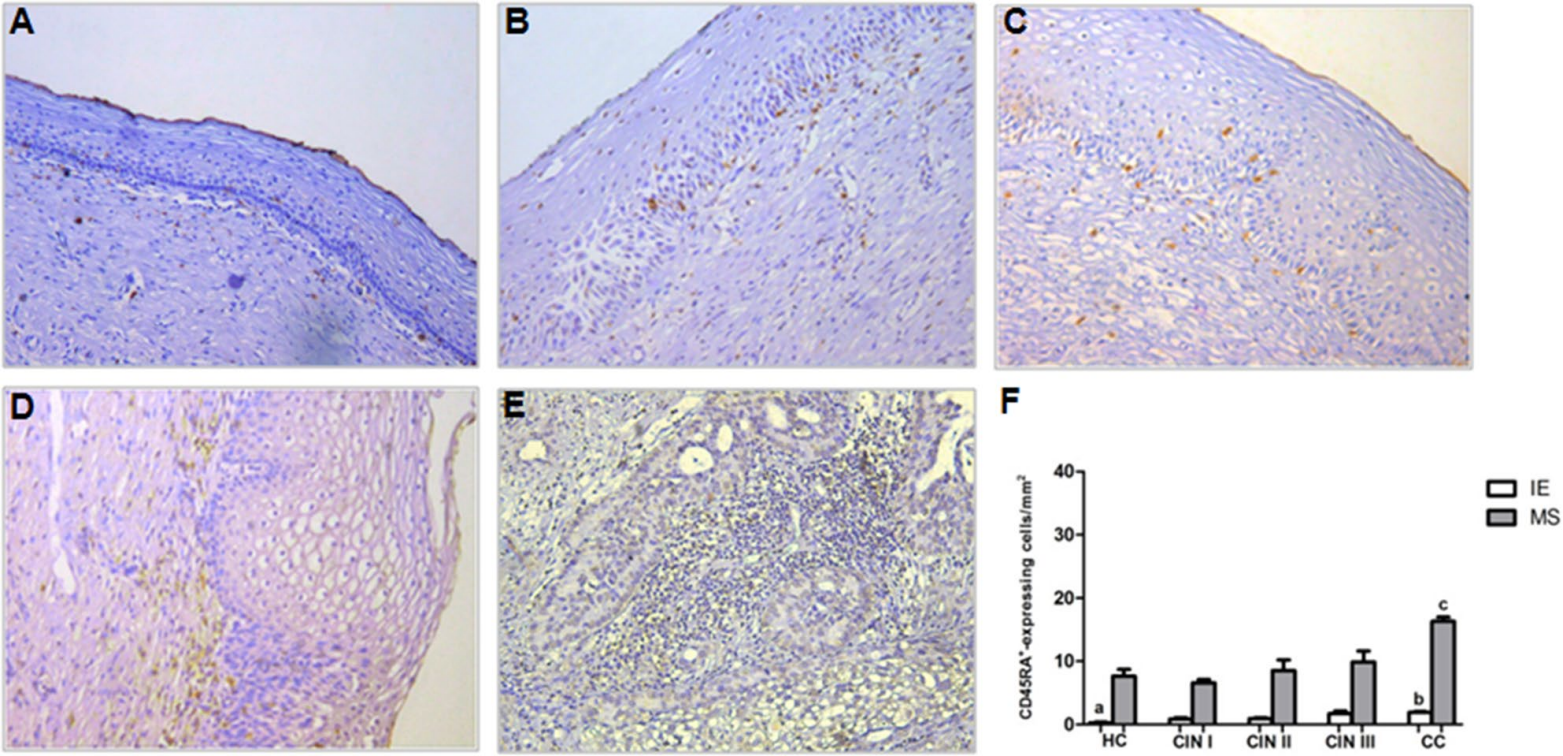
Immunohistochemical staining showing CD45RA^+^-expressing cells in uterine cervix tissues in IE and MS sites from (**A**) HC, (**B**) CIN I, (**C**) CIN II, (**D**) CIN III, and (**E**) CC. Original magnification = × 20, (**F**) represents the means ± SD of CD45RA expressing cell counts among the groups. ^a^*p* < 0.01, HC versus CIN III; *p* < 0.001, HC versus CC. ^b^*p* < 0.001, CC versus CIN I. ^c^*p* < 0.001, CC versus HC, CIN I, II and III. *P* value was calculated using the nonparametric Kruskal–Wallis test and post-test of Dunns for multiple comparisons.

We first performed a descriptive cell distribution analysis in the intraepithelial (IE) and marginal stroma (MS) areas of cervical biopsies. These areas were selected because epithelium is the preferential site of HPV infection and neoplastic differentiation, while stroma is the marginal area adjacent to the lesion site where there is inflammatory cell infiltration. CD45RA^+^ expressing cells distribution in IE and MS areas among groups is shown in Fig. 1. A significant increase in CD45RA^+^ expressing cells’ distribution was observed in both IE and MS areas from the CC group, compared to CIN I (*p* < 0.001, in both areas) and HC (*p* < 0.001, in both areas) groups. Besides, MS of the CC group presented a higher CD45RA^+^ expressing cells distribution when compared to CIN II (*p* < 0.001) and CIN III (*p* < 0.001). A threefold increase in number of CD45RA expressing cells in CIN III (*p* < 0.01) compared to HC group was observed only in IE area. The frequency of CD45RA^+^ in the MS of CIN III patients was very heterogeneous and even then, these results pointed to an increased distribution of these cells according to cervical lesion severity.

**Figure 2:**
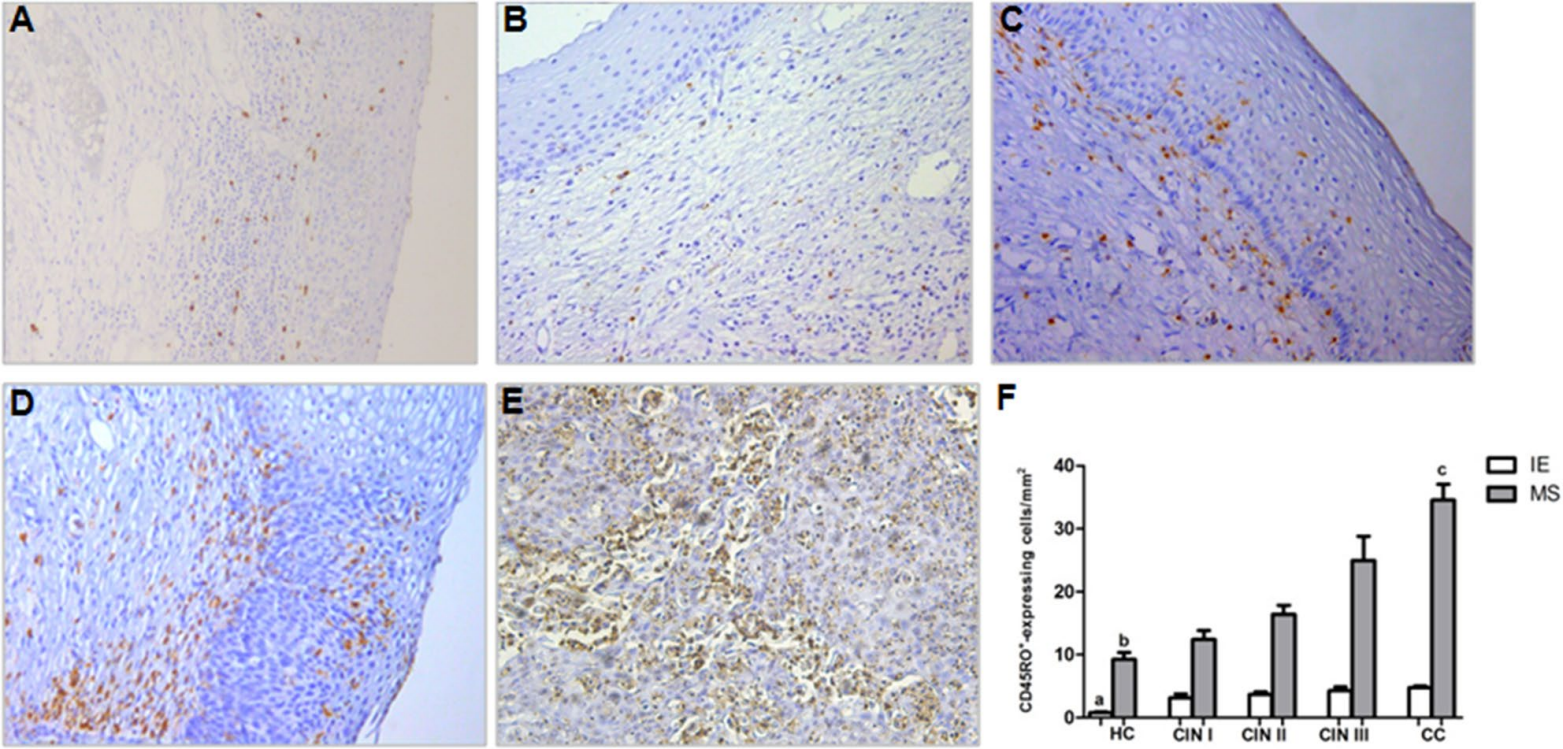
Immunohistochemical staining showing CD45RO^+^-expressing cells in uterine cervix tissues in IE and MS sites from (**A**) HC, (**B**) CIN I, (**C**) CIN II, (**D**) CIN III, and (**E**) CC. Original magnification = × 20, (**F**) represents the means ± SD of CD45RO expressing cell counts among the groups. ^a^*p* < 0.05, HC versus CIN I; *p* < 0.001, HC versus CIN II, III and CC. ^b^*p* < 0.01, HC versus CIN III. ^c^*p* < 0.001, CC versus CIN I and HC; *p* < 0.01, CC versus CIN II. *P* value was calculated using the nonparametric Kruskal–Wallis test and post-test of Dunns for multiple comparisons.

Regarding the distribution of CD45RO^+^ expressing cells (Fig. 2), CC and CIN III group have an increased frequency compared to HC group in IE (*p* < 0.001, in both areas), and MS (*p* < 0.001 and *p* < 0.01 respectively) areas. Only in MS area, CC presented a higher distribution than CIN II (*p* < 0.01) and CIN I (*p* < 0.001). In the IE area, CIN I and CIN II patients showed an increased frequency (*p* < 0.05 and *p* < 0.001, respectively) of these cells compared to HC groups. *P* values related to the distribution of CD45RA^+^ and CD45RO^+^ cells were calculated using the nonparametric Kruskal–Wallis test and post-test of Dunns for multiple comparisons.

### Distribution of CCL20^+^ and CCR6^+^ cells decrease according to cervical lesion severity

**Figure 3:**
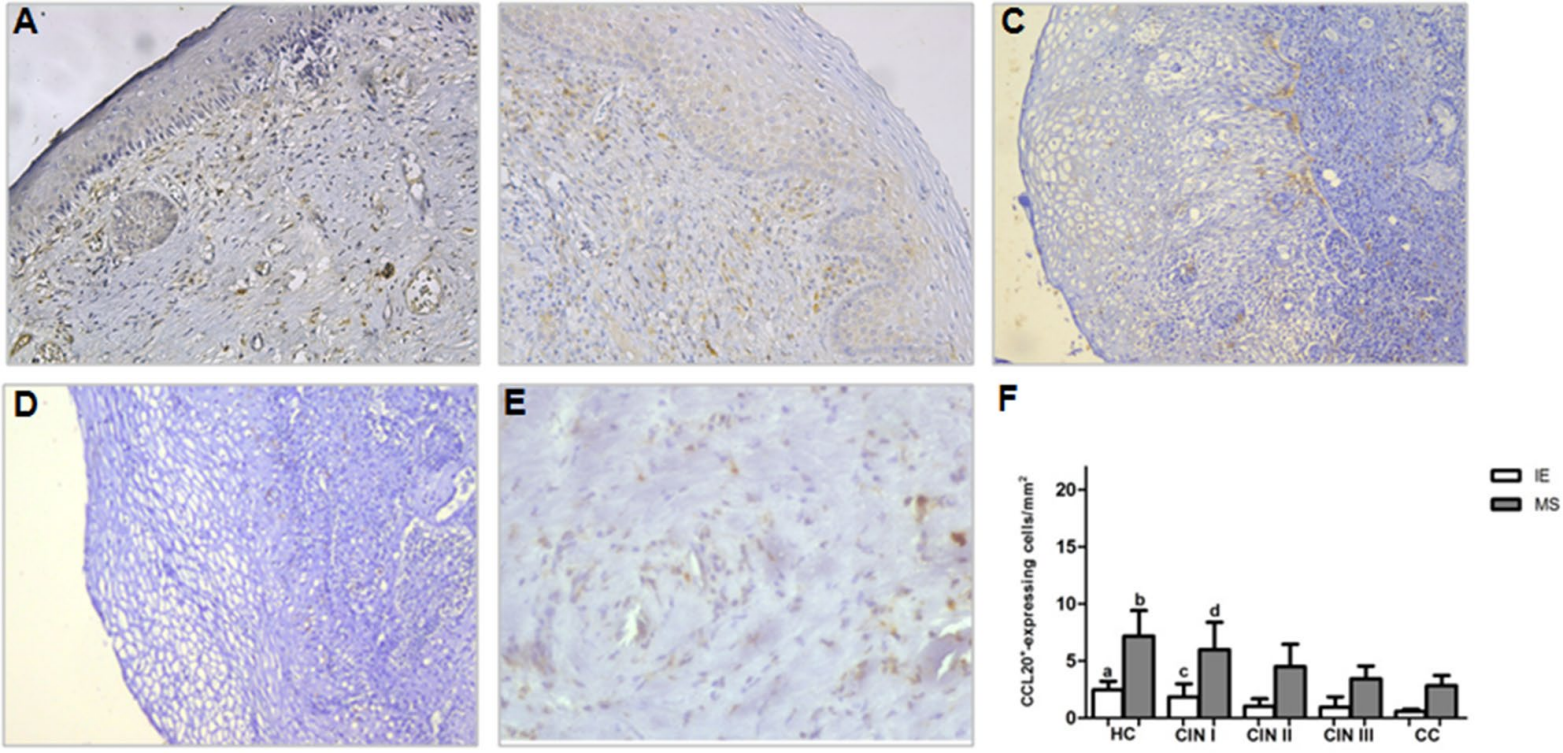
Immunohistochemical staining showing CCL20^+^-expressing cells in uterine cervix tissues in IE and MS sites from (**A**) HC, (**B**) CIN I, (**C**) CIN II, (**D**) CIN III, and (**E**) CC. Original magnification = × 20, (**F**) represents the means ± SD of CCL20 expressing cell counts among the groups. ^a^*p* < 0.01, HC versus CIN II; *p* < 0.001, HC versus CIN III and CC. ^b^*p* < 0.05, HC versus CIN II; *p* < 0.001, HC versus CIN III and CC. ^c^*p* < 0.05, CIN I versus CIN III and CC. ^d^*p* < 0.01, CIN I versus CIN III; *p* < 0.001 CIN I versus CC. *P* value was calculated using the nonparametric Kruskal–Wallis test and post-test of Dunns for multiple comparison.

To determine the inflammatory cell migration into IE from MS, CCL20^+^ and CCR6^+^ expressing cells were identified. Interestingly, we observed a reduction of CCL20^+^ expressing cells’ distribution according to lesion severity (Fig. 3). CC group had a significant decrease in CCL20^+^ expressing cells distribution in both IE and MS areas, markedly when compared to CIN I (*p* < 0.05 and *p* < 0.001, respectively) and HC group (*p* < 0.001, in both areas). Similarly to CC, CIN III presented a reduction in these cells’ distribution in IE and MS areas when compared to HC group (*p* < 0.001 in both areas) and CIN I (*p* < 0.05 and *p* < 0.01, respectively). CIN II presented a reduction in these cells when compared only to the HC group in IE (*p* < 0.01) and MS (*p* < 0.05).

**Figure 4:**
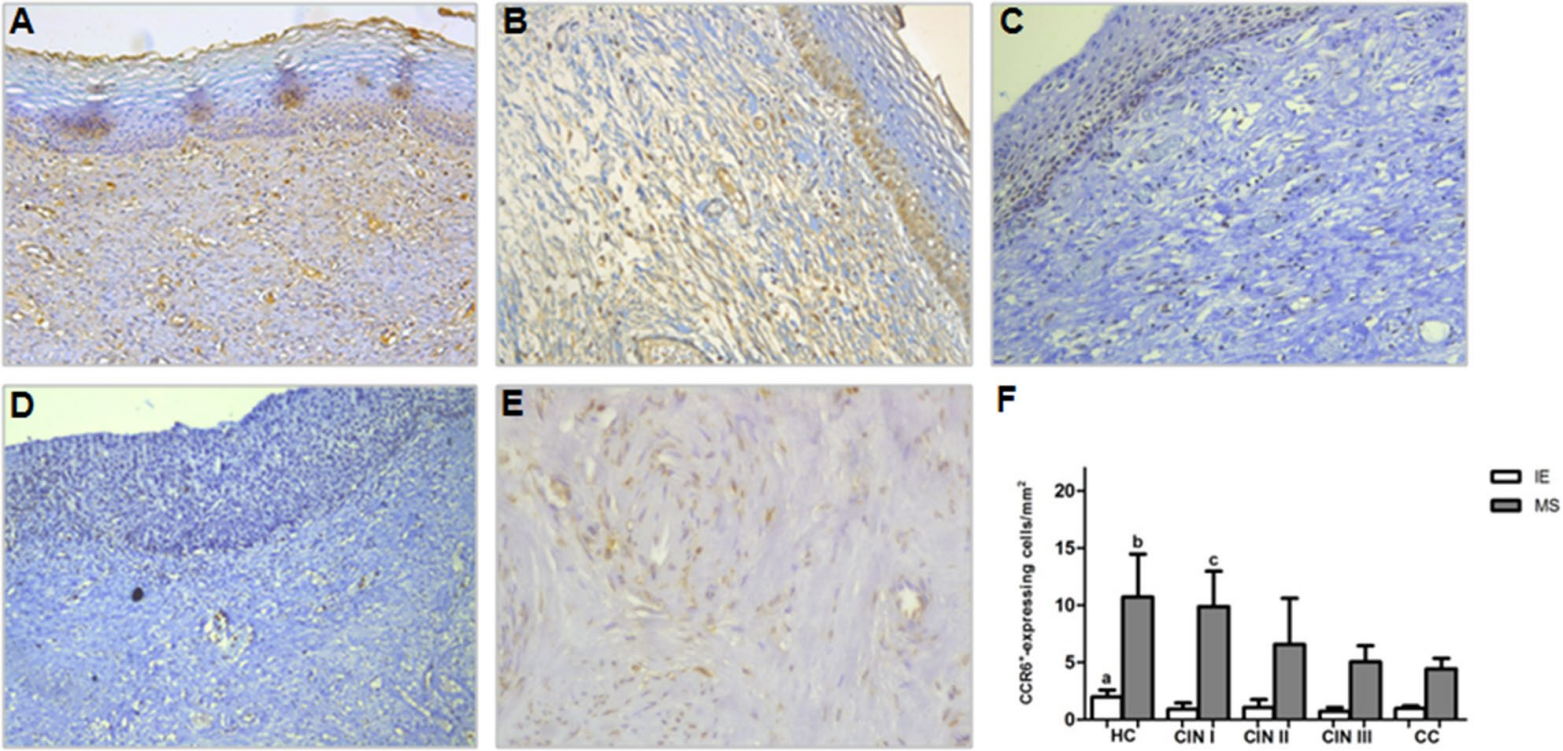
Immunohistochemical staining showing CCR6^+^-expressing cells in uterine cervix tissues in IE and MS sites from (**A**) HC, (**B**) CIN I, (**C**) CIN II, (**D**) CIN III, and (**E**) CC. Original magnification = × 20, (**F**) represents the means ± SD of CCL20 expressing cell counts among the groups. ^a^*p* < 0.05, HC versus CC; *p* < 0.01, HC versus CIN II; *p* < 0.001, HC and CIN I and CIN III. ^b^*p* < 0.05, HC versus CIN II; *p* < 0.001, HC versus CIN III and CC. ^c^*p* < 0.01, CIN I versus CIN III; *p* < 0.001, CIN I and CC. *P* value was calculated using the nonparametric Kruskal–Wallis test and post-test of Dunns for multiple comparison.

CCR6^+^ expressing cells showed similar distribution as CCL20^+^ ones (Fig. 4), with the CC group presenting lower expressing-cell frequency when compared to the HC in IE (*p* < 0.05) and MS areas (*p* < 0.001), and with CIN I only in MS areas (*p* < 0.001). CIN III and II presented a reduced cell dispersion when compared to HC in IE (*p* < 0.001 and *p* < 0.01, respectively) and MS (*p* < 0.001 and *p* < 0.05, respectively) areas. These results indicate that HPV-infection may interfere in CCR6^+^ expression from cervical lesions, impairing the inflammatory cell migration to HPV infection site. *P* values related to the distribution of CCL20^+^ and CCR6^+^ cells were calculated using the nonparametric Kruskal–Wallis test and post-test of Dunns for multiple comparisons.

We performed a multiple linear regression analysis to determine the effect of clinical and environmental variables on the number of CD45RA^+^, CD45RO^+^, CCL20^+^ and CCR6^+^ expressing cells in both IE and MS sites. Variables such as age, tobacco use, alcohol consumption and number of abortions, as represented in Tables 2 and 3, were selected since they presented a *p* < 0.20 in comparison between groups (Table 1).

**Table 2:** Multiple linear regression analysis of CD45RA^+^ and CD45RO^+^ expressing cells.

	CD45RA^+^		CD45RO^+^	
	IE*	MS*	IE*	MS*
	Coef. (95% CI) *p* value	Coef. (95% CI) *p* value	Coef. (95% CI) *p* value	Coef. (95% CI) *p* value
Age (in years)	0.027 (− 0.0002 to 0.0534) 0.052	**0.165 (0.0137 to 0.3153) 0.033**	0.022 (− 0.0306 to 0.0751) 0.402	**0.383 (0.0704 to 0.6946) 0.017**
Tobacco use	0.061 (− 0.5468 to 0.6682) 0.842	0.831 (− 2.585 to 4.248) 0.628	0.738 (“ − 0.4585 to 1.934”) 0.222	5.356 (− 1.715 to 12.43) 0.135
Alcohol consumption	0.178 (− 0.4026 to 0.7576) 0.543	− 0.371 (− 3.633 to 2.892) 0.821	− 0.238 (− 1.381 to 0.9042) 0.678	− 0.573 (− 7.325 to 6.179) 0.866
Number of abortions	− 0.191 (− 0.5668 to 0.1857) 0.315	0.136 (− 1.980 to 2.252) 0.898	− 0.705 (− 1.446 to 0.0360) 0.062	− 0.826 (− 5.205 to 3.553) 0.707

*IE intraepithelial, *MS* marginal stroma.

The bold values mean statistical difference in the multiple linear regression analysis.

**Table 3:** Multiple linear regression analysis of CCL20^+^ and CCR6^+^ expressing cells.

	CCL20^+^		CCR6^+^	
	IE*	MS*	IE*	MS*
	Coef. (95% CI) *p* value	Coef. (95% CI) *p* value	Coef. (95% CI) *p* value	Coef. (95% CI) *p* value
Age (in years)	− 0.004 (− 0.0254 to 0.0184) 0.750	− **0.057 (**− **0.1022 to** − **0.0114) 0.015**	0.008 (− 0.0035 to 0.0195) 0.170	− **0.086 (**− **0.1592 to** − **0.0125) 0.023**
Tobacco use	− **0.536 (**− **1.031 to** − **0.0410) 0.034**	− 0.9961 (− 2.025 to 0.0326) 0.058	− 0.095 (− 0.3558 to 0.1665) 0.471	− **1.88 (**− **3.546 to** − **0.2219) 0.027**
Alcohol consumption	0.024 (− 0.4493 to 0.4965) 0.921	0.0980 (− 0.8844 to 1.080) 0.843	0.024 (− 0.3558 to 0.1665) 0.848	− 0.790 (− 2.377 to 0.7973) 0.324
Number of abortions	0.047 (− 0.2599 to 0.3534) 0.762	**0.8951 (0.2581 to 1.532) 0.007**	0.081 (− 0.2746 to 0.0488) 0.168	**1.27 (0.2388 to 2.297) 0.017**

*IE intraepithelial, *MS* marginal stroma.

The bold values mean statistical difference in the multiple linear regression analysis.

The variable age had a positive effect on the number of CD45RA^+^ and CD45RO^+^ expressing cells in MS, inducing an increase of 0.165 (*p* = 0.033) and 0.383 (*p* = 0.017) in number of cells per each year of patients’ lives, respectively (Table 3). However, the number of CCL20^+^ expressing cells was negatively affected in MS by age (coef. -0.057, *p* = 0.015) and in IE by tobacco use (coef. -0.536, *p* = 0.034). Interestingly, the variable number of abortions had a positive effect on CCL20^+^ expressing cells in MS (coef. 0.8951, *p* = 0.007).

The variables age and tobacco use had a negative effect in the number of CCR6^+^ expressing cells in MS (coef. − 0.086, *p* = 0.023, and coef. − 1.88, *p* = 0.027, respectively). In contrast, for each abortion, there is an increase of 1.27 in the number of CCR6^+^ expressing cells in the MS (coef. 1.27, *p* = 0.017).

### Relative ratio among inflammatory cells in cervical lesion

**Figure 5:**
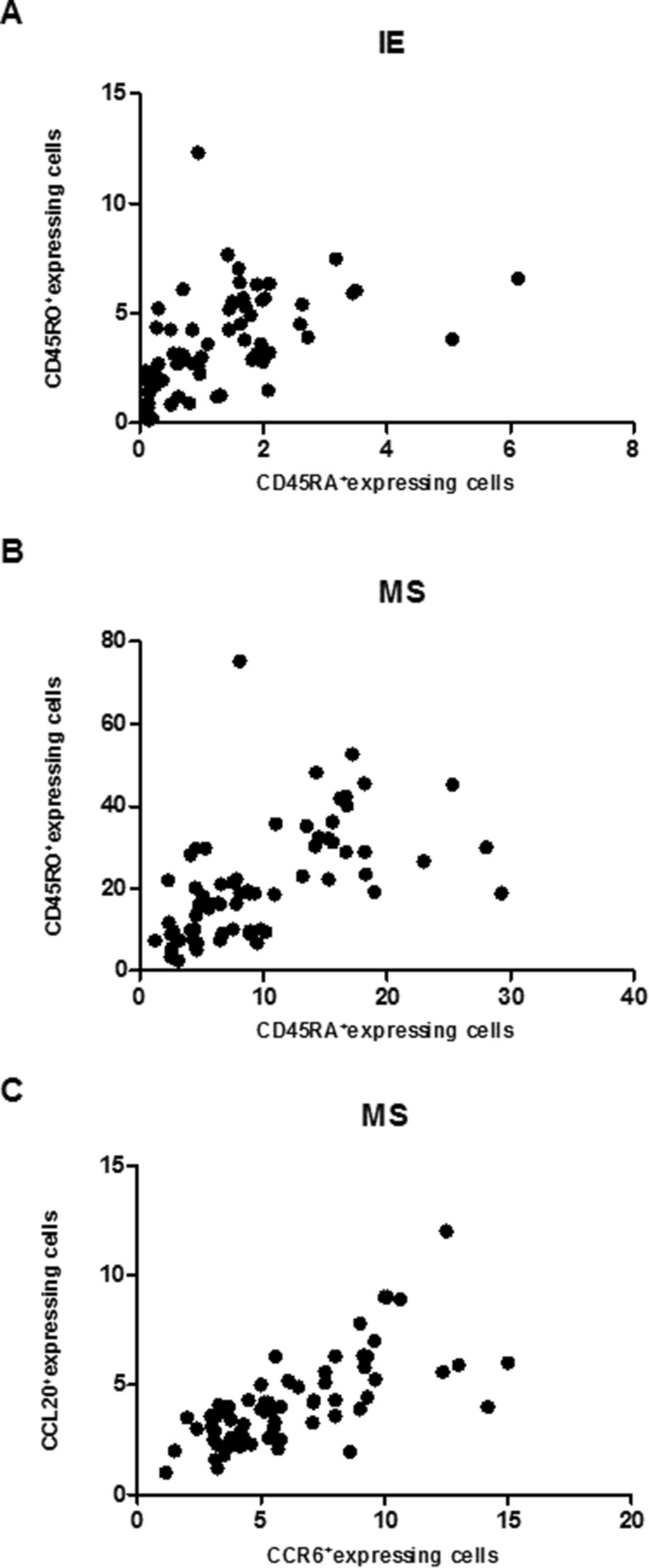
Correlation among inflammatory cells in cervical lesion. (**A**) CD45RA^+^ and CD45RO^+^ cells distribution in IE and (**B**) MS. (**C**) CCL20^+^ and CD45RO^+^ cells distribution in MS. Spearman’s rank correlation was used to identify association between the cell markers. *P* value was calculated using the Spearman test.

A moderate positive correlation between CD45RA^+^ and CD45RO^+^ cells’ distribution in IE (ρ = 0.61, *p* < 0.001—Fig. 5A) and in MS (ρ = 0.67, *p* < 0.001—Fig. 5B) and strong positive correlation between CCL20^+^ and CCR6^+^ cells’ distribution only in MS (ρ = 0.74, *p* < 0.001—Fig. 5C) was observed. However, a weak negative correlation between CCL20^+^ and CD45RO^+^ cells (ρ = − 0.37, *p* < 0.01) was reported (data not shown). No correlation was seen between CCL20^+^ and CD45RA^+^ cells (*p* > 0.05). *P* values were calculated using the Spearman´s correlation test.

### ‘Immunoscore’ profile as an indicator of SIL and CC progression

**Figure 6:**
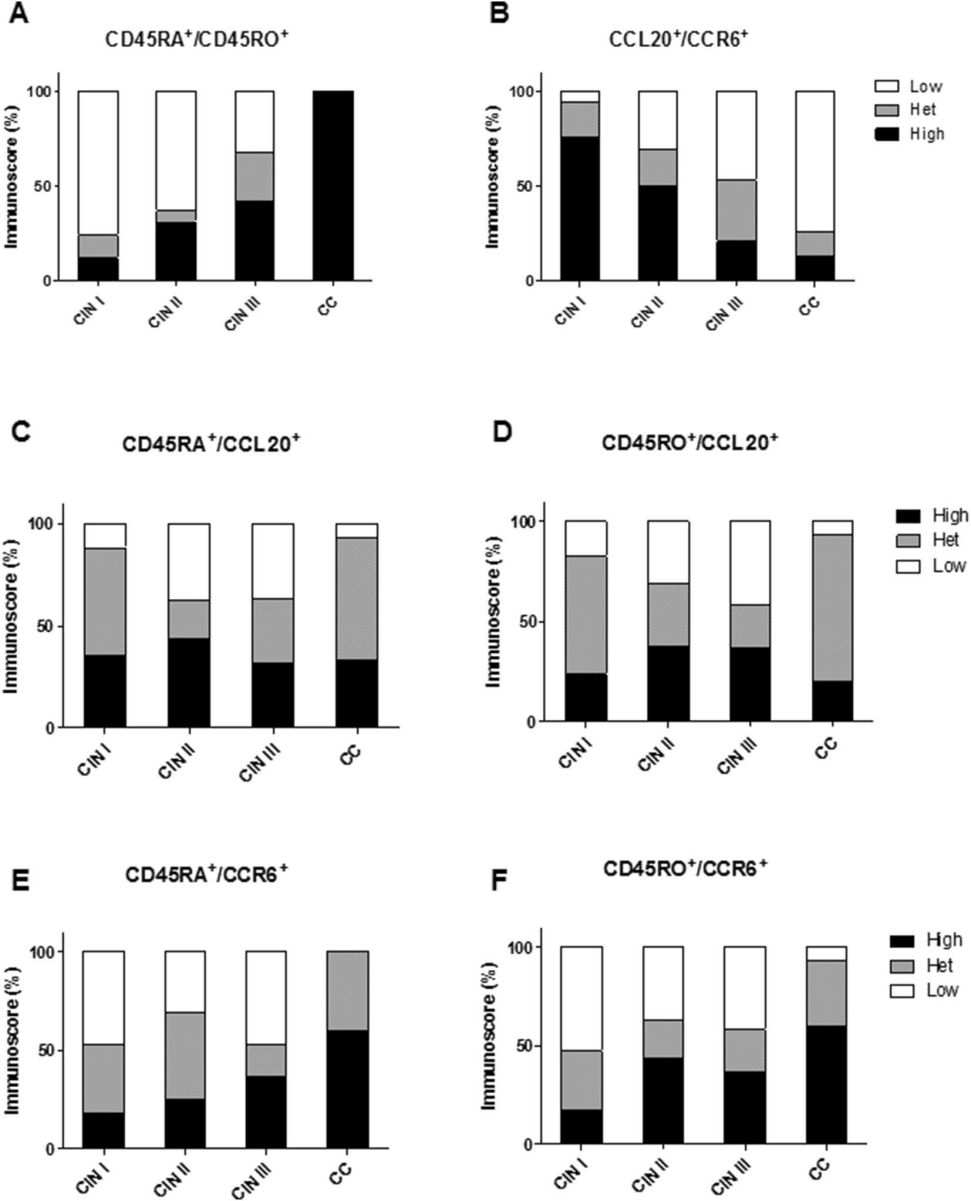
Immunoscore profile for (**A**) CD45RA^+^/CD45RO^+^ and (**B**) CCL20^+^/CCR6^+^, (**C**) CCL20^+^/CD45RA^+^, (**D**) CCL20^+^/CD45RO^+^, (**E**) CCR6^+^/CD45RA^+^ and (**F**) CCR6^+^/CD45RO^+^ as an indicators of SIL and CC progress.

To assess the dynamic distribution of the immune cells in the cervical lesions’ microenvironment, we analyzed the ‘immunoscore’ profile in CIN and CC groups. According to lesion severity, an increasing number of women showed high ‘immunoscore’ for CD45RA^+^/CD45RO^+^ in the CC group when compared to CIN I (, χ^2^ = 24.9, *p* = 0.0006), CIN II (χ^2^ = 15.98, *p* = 0.0006) and CIN III groups (χ^2^ = 12.84, *p* = 0.0096, Fig. 6A). Interestingly, an inverse CCL20^+^/CCR6^+^ ‘immunoscore’ profile was observed in the CC group, when compared to CIN I (χ^2^ = 16.54, *p* = 0.002), CIN II (χ^2^ = 6.02, *p* = 0.049) and CIN III (χ^2^ = 12.09, *p* = 0.01) (Fig. 6B).

An individual CD45RA^+^/CD45RO^+^ and CCL20^+^/CCR6^+^ ‘immunoscore’ analysis was performed for each patient to determine if these biomarkers could be used to distinguish disease grade. High CD45RA^+^/CD45RO^+ ‘^immunoscore’ was identified in only one patient with CINI II (6.2%) and two patients with CIN III (10.5%). Aside from those in the CC group, suggesting that this inflammatory profile may have a pivotal role in the disease severity. However, when low CD45RA^+^/CD45RO^+^ and high CCL20^+^/CCR6^+^ ‘immunoscores’ were analyzed in combination, we observed 5 CIN II and 2 CIN III patients with this profile, similar to that described in the CIN I group. Interestingly, these women did not present any inflammatory reaction in their cervical histopathology.

No statistical difference was observed when we used the CD45RA^+^/CCL20^+^, CD45RO^+^/CCL20^+^ and CD45RO^+^/CCR6^+^ combined ‘immunoscores’ as biomarkers (Fig. 6C, D, F). However, when CD45RA^+^/CCR6^+^ ‘immunoscore’ was used, we observed an increasing number of CC women presenting a high ‘immunoscore’ compared to CIN I (*p* < 0.01.043, χ^2^ = 10.9), CIN II (*p* < 0.05, χ^2^ = 7.0) and CIN III (*p* < 0.01, χ^2^ = 9.92, Fig. 6E).

Because the clinical and environmental variables showed influence in the number of CD45RA^+^, CD45RO^+^, CCL20^+^ and CCR6^+^ expressing cells, we performed a logistic regression analysis to determine the effect of these variables on the ‘immunoscore’ analysis. Only age had a positive effect in CD45RA^+^/CD45RO^+^ ‘immunoscore’ in low versus high ‘immunoscore’ (Table 4). To an increment of one year in the age of the patients, there is an increase of 1.09 times in the chance to the patient to be classified as high CD45RA^+^/RO^+^ ‘immunoscore’ (OR 1.09, *p* = 0.01). The logistic regression analysis of CCL20/CCR6 showed negative effect of age and tobacco use, and a positive effect of the number of abortions in low versus het ‘immunoscore’ (OR 0.90, *p* = 0.03; OR 0.13, *p* = 0.049; and OR 2.81, *p* = 0.04, respectively, Table 5). In contrast, only tobacco use had a negative effect in low versus high CCL20^+^/CCR6^+^ ‘immunoscore’ (OR 0.24, *p* = 0.04).

**Table 4:** Logistic regression analysis in CD45RA^+^/CD45RO^+^ ‘immunoscore’ data.

	CD45RA^+^/CD45RO^+^		
	Low × het	Low × high	Het × high
	OR (95% CI) *p* value	OR (95% CI) *p* value	OR (95% CI) *p* value
Age (in years)	1.05 (0.93–1.19), 0.45	**1.09 (1.03–1.18), 0.01**	0.98 (0.87–1.09), 0.72
Tobacco use	7.85 (0.88–207.1), 0.11	2.17 (0.64–7.97), 0.22	0.10 (0.00–1.26, 0.11)
Alcohol consumption	0.16 (0.02–0.99), 0.067	0.83 (0.25–2.72), 0.75	10.46 (0.93–259.4), 0.09
Number of abortions	0.20 (0.01–0.97), 1.19	0.67 (0.29–1.42), 0.31	3.22 (0.47–68.05), 0.31

The bold values mean statistical differences between the groups.

**Table 5:** Logistic regression analysis in CCL20^+^/CCR6^+^ ‘immunoscore’ data.

	CCL20^+^/CCR6^+^		
	Low × het	Low × high	Het × high
	OR (95% CI) *p* value	OR (95% CI) *p* value	OR (95% CI) *p* value
Age (in years)	**0.90 (0.81–0.98), 0.03**	0.94 (0.88–1.00). 0.06	1.02 (0.94–1.10), 0.68
Tobacco use	**0.13 (0.01–0.87), 0.049**	**0.24 (0.05–0.87), 0.04**	0.91 (0.21–3.78), 0.90
Alcohol consumption	1.85 (0.39–10.42), 0.46	0.79 (0.23–2.75), 0.71	0.83 (0.20–3.22), 0.79
Number of abortions	**2.81 (1.13–9.01), 0.04**	2.10 (0.71–6.97), 0.19	0.56 (0.22–1.29), 0.19

The bold values mean statistical differences between the groups.

## Discussion

The interplay between cancer and immune cells is a major determining factor in cancer progression and may be a powerful prognostic marker for carcinogenesis. The HPV cervical inflammatory process and subsequent development of malignant lesions are induced directly or indirectly by a complex system composed of the interaction between HPV oncogenes and host factors secreted by keratinocytes, immune and stromal cells. In our study, we investigated the possible correlation between intralesional immune cell profile and HPV-associated cervical lesion severity.

First of all, we identified the CD45RA^+^, CD45RO^+^, CCL20^+^ and CCR6^+^ expressing cell distribution in cervical lesions and CC. To our knowledge, there are no previous studies reporting in situ distribution of these markers in the uterine cervix in different grades of cervical lesions and cancer. However, several reports using peripheral blood mononuclear cells (PBMC) showed a difference between naïve (CD45RA^+^) and memory T cell (CD45RO^+^) populations in HPV infection^6,25,26^. Our results showed increased CD45RA^+^ frequency in the IE area in patients with premalignant lesions (CIN III) compared to HC. Previous reports using cervical cells obtained through scraping showed high rates of CD45RA^+^ T cells in the epithelial region of regressing lesions^27^. This association was also identified in PBMC from patients with head and neck squamous cell carcinoma (HNSCC), compared to HC participants, indicating that naïve cell population was increased in accordance with the lesion severity^28^. Pita-Lopez^29^ and colleagues reported reduction of CD45RA^+^ T cells in PBMC from CIN I patients compared to HC and their association with the HPV persistent infection.

The analysis of CD45RO + T cells demonstrated that the distribution throughout the cervical tissue was more frequent in the stroma area in all groups, remarkably in CIN III patients, suggesting increased number of CD45RO^+^ cells in this region can be indicative of a worst prognosis. The increased presence of this cell population in combination with other markers, such as CD4^+^, CD8^+^ and CD27^+^ in different stages of CIN may be related to persistent lesions, an important feature for the premalignant lesions’ progression and consequent CC development^30^. A similar profile was described by Monnier-Benoit^31^ and colleagues when comparing CD45RO^+^ T cells infiltrate in participants diagnosed with CIN I, CIN II and CIN III, and invasive carcinoma. Interestingly, Maluf et al.^32^ showed a lack of association between lesion recurrence and increased CD45RO^+^ T cells in a longitudinal study in volunteers before and after conization, that could indicate association with cervical lesion progression but not cure. Unfortunately, we did not distinguish CD4^+^ and CD8^+^ T cells expressing the memory phenotype, however in previous works, we observed that the CD8^+^ T cells were remarkably present in high-grade squamous intraepithelial lesion groups^7^. These results are in agreement to Monnier-Benoit and colleagues^31^, suggesting that CD4^+^ T cell frequency may be indicative of CIN I regression, while the CD8^+^ T frequency points to the lesion severity.

Our results are consistent with the literature and showed association with CD45RO^+^ cell increase and lesion severity. However, this profile may not be associated with other tumor types. Berghoff and colleagues^33^ demonstrated an association between high CD45RO^+^ tumor-infiltrating lymphocyte (TIL) density and a favorable overall survival in brain metastasis. In situ studies using double/ triple cell markers could identify the location of T-cell subpopulations and their stages of maturation during progression from pre-malignant lesion to CC. It is well established that CD8^+^ T cells are the major intra-lesion and intra-tumor T cells, especially in advanced lesions^34^. However, our main goal was to identify, using CD45RA and CD45RO markers, as predictors of CIN outcome to CC, and our results support this hypothesis in part. The ability of effector-memory T cells to recall previously known antigens leads to a protective response. Following a primary exposure to antigen, memory T cells disseminate and are maintained for long periods after cancer development^35^. The trafficking properties and the long-lasting antitumor capacity of memory T cells could result in long-term immunity in human cancer.

The CCL20 chemokine is constitutively expressed in a wide variety of cells and tissues. CCR6, as the single receptor with high affinity for CCL20, is primarily expressed on the surface of Langerhans cells (LC), dendritic cells and activated T- and B-cells. The CCL20 and CCR6 axis plays a crucial role in the process of LC gathering and chemotaxis in cervical epithelial tissue^36–38^. We observed a decrease in the number of cells expressing CCL20 in the IE, site of HPV infection, and MS area according to lesion severity. The low-risk (HPV 6 and 11) and high-risk (HPV 16) HPV E6 and E7 oncoproteins may influence CCL20 transcription in infected keratinocytes in vitro^37,38^, indicating that HPV may be negatively modulating the expression of this chemokine in the epithelium by blocking the migration of inflammatory cells, such as LCs to the lesion site. A high number of CCL20^+^ cells were also identified in the stroma region of patients with CIN III^10^. In vitro studies have shown that infected keratinocytes can induce a strong expression of CCL20 by stromal fibroblasts, which could possibly explain the increased frequency of these cells in this region^10^. Similarly, increased expression of microRNA21, induced by HPV16 E6 and E7, may lead to decreased CCL20 expression and tumor progression and carcinogenesis^39^. These results might explain one of the mechanisms used by HPV to evade the immune system. Recently, Scagnolari and collaborators^40^ showed that female C57BL/6J mice are susceptible to a transient papillomavirus cervicovaginal infection, and mice deficient in select genes involved in innate immune responses, as CCR6, are susceptible to persistent infection with variable manifestations of histopathological abnormalities. A better understanding of mechanisms of early viral clearance and development of approaches to induce clearance will be important for a better understanding of CC’s natural history and, possibly, contributing to its prevention and treatment.

An ‘immunoscore’ has been used to determine the immune microenvironment profile in cancers, based on cell population counts in different sites to infer the possible role of these cells in the carcinogenic process. Galon et collaborators^41^ suggested that ‘immunoscore’ could provide a more accurate clinical prognosis compared to that of TNM staging, that is the most widely used method to predict the clinical outcomes of cancer patients. However, patients with the same TNM staging may present a variety of clinical responses. This classification method focuses only on the tumor characteristics, but not on the immune response present in these tumors. The relationship between tumor cells and infiltrating immune cells was neglected^42^. Thus, it is not enough to obtain an accurate outcome prediction by TNM staging alone. Immune status may play pivotal roles in tumor progression and prognosis^43^. As a result, studies have proposed the ‘immunoscore’ to predict clinical outcomes of cancer^44–46^. In CC, there are a few studies about ‘immunoscore’, but none using CD45RA^+^/CD45RO^+^ and CCL20^+^/CCR6^+^ as possible prognostic markers. CD45RA^+^ and CD45RO^+^-expressing cells have been extensively explored in many cancers, such as colon^47^, rectal^48^, lung^49^, renal hepatocellular^50^ and head and neck^51^ squamous cell carcinoma. Because of that, they would be valid candidates to be used as ‘immunoscore’ markers in cervical cancer in further studies.

A high immune infiltrate is associated with better clinical outcomes, or lesion regression, when it is brief and well controlled. However, the chronic HPV infection and misled immune responses in the local immune microenvironment play a critical role during the progression of precancerous lesions to invasive cancer^52–54^. Immune evasion is an important cause of persistent HPV infection. There is no detectable inflammatory reaction at the early stages as well as activation of the innate immune system^55^. Once established, the persistent infection triggers changes in the secretion of inflammatory cytokines, which in turn leads to immune cell infiltration^56^. In fact, we observed increasing inflammatory infiltrate according to CIN severity and CC. However, the role of HPV infection in the induction of chronic inflammation and the link between chronic inflammation and HPV-induced CC carcinogenesis remains controversial.

Other biomarkers have been studied and correlated with cervical carcinogenesis. Van Zummeren^23^ investigated the accuracy and reproducibility of a scoring system for CIN I-III based on Ki-67^+^/p16^ink4a^ biomarkers. Ki-67 is an indicator of cellular proliferation, whereas diffuse p16^ink4a^ staining occurs when it is overexpressed as a result of functional inactivation of retinoblastoma protein by the HPV E7 protein. They found that higher ‘immunoscores’ were more frequently observed in CIN III patients than in those with CIN I. Corroborating these results, Kremer and collaborators observed that Ki-67 and p16^ink4a^ scores increased with increasing CIN grade in HIV-infected women.

**Table 6:** HPV primers sequence.

Primers set	Sequences	Amplification size (PB*)
MY09/11	CGT CC**M** A**RR** GGA **W**AC TGA TC	450
	GC**M** CAG GG**W** CAT AA**Y** AAT GG	
GH20/PC04	GAA GAG CCA AGG ACA GGT AC and CAA CTT CAT CCA CGT TCA CC	268
	CAA CTT CAT CCA CGT TCA CC	

*M = A + C; R = A + G; W = A + T; Y + C + T*pb—pairs bases.

Chen and collaborators^24^ investigated the CD8^+^ T cells and programmed cell death receptor 1 (PD-1) and its ligand (PD-L1) expressions and their potential role in ‘immunoscore’ TNM classification of CC. They observed that patients with PD-L1^+^ and PD-1^high^ in immune cells had poorer overall survival and disease-free survival. However, PD-L1^+^ in tumor cells that infiltrated more CD8^+^ T cells were related to better overall survival and disease-free survival. These immune factors can be independent predictors for prognoses.

Tumor-infiltrating lymphocytes (TILs) presence has been correlated with positive patient outcome in many tumor types, including colorectal cancer, melanoma, breast carcinoma, urinary bladder, prostate, renal cell, head and neck, lung, esophageal, gastric, pancreatic, hepatocellular and ovarian carcinoma^41,42,57–59^. Although the prognostic significance of the various TILs subpopulations, their density and location may vary according to the tumor type and stage^51^.

We determined the effect of clinical and environmental variables in the expression of cellular markers and ‘immunoscore’. The age, tobacco use, alcohol consumption and number of abortions showed an influence in the number of cells expressing CD45RA^+^, CD45RO^+^, CCL20^+^ and CCR6^+^ markers in IE and MS, as well as in the ‘immunoscore’ comparisons. It is described that HPV infection is extremely common in young women in their first decade of sexual activity. Persistent and high-grade HPV infections are established, typically within 5–10 years, in less than 10% of new infections. Invasive cancer arises after many years of infection, even decades, in a minority of women with precancerous lesions, with a peak or plateau risk at 35–55 years old^60^. So we hypothesize that the age could have no direct influence in the expression of these markers, but it is linked to the slowly time of CC progression.

The others clinical and environmental variables had no effect on the CD45RA^+^ and CD45RO^+^ expressing cells distribution as well as CD45RA^+^/CD45RO^+^ ‘immunoscore’, showing no contribution of tobacco use, alcohol consumption or abortion in these markers and ‘immunoscore’ classification. On the other hand, these variables showed effect on the distribution of CCL20^+^ and CCR6^+^ expressing cells and the CCL20^+^/CCR6^+^ ‘immunoscore’. Number of CCL20^+^ expressing cells was negatively affected in IE by tobacco use. Increased levels of tobacco substances, such as nicotine and cotinine, were found in the cervical mucus and prostate sperm fluids of smokers and passive smokers^61^. This indicates that they reach the uterine cervix and lead to increased modification of DNA in the cervical epithelium, suggesting biochemical evidence of smoking as a cause of cervical cancer^62^. Besides, Siokos et al.^63^ demonstrated that nicotine had an effect in overall damage of the immune system as well as the reduction of cervical self-defense making it more vulnerable to the carcinogenic nature of HPV.

Despite the statistically significant effect, we hypothesized that these clinical and environmental variables could have minor biological contribution on the distribution of cell markers and their related ‘immunoscore’. Indeed, these variables contribute for a little increase or decrease in cell markers expression, compared to overall mean. However, the effect of alcohol consumption and number of abortions in the immune response of HPV infection as well as in the expression of these cell markers have to be elucidated.

In HPV-induced lesions, as CIN and CC, there is now solid evidence for a stage-specific interplay between virally-infected keratinocytes and the local immune microenvironment that can determine the course of disease. Novel diagnostic tools, including ‘immunoscores’, might allow the discrimination of non-progressors and progressors precursor lesions during the HPV infection. The E6 and E7 oncoproteins of hrHPV groups have a fundamental role in precursor lesion development. As described in this study, our results demonstrated, individually, an increase in the CD45RA^+^ and CD45RO^+^ cell dispersion, and a decrease in the CCL20^+^ and CCR6^+^ cell dispersion as the cervical lesions severity. Based on these results, we decided to evaluate the prognostic value of ‘immunoscore’ with these markers, and suggested a pattern in the cervical inflammatory process during the HPV infection outcome with high CD45RA^+^/CD45RO^+^ and low CCL20^+^/CCR6^+^ ‘immunoscore’, especially in high grade lesions. We expect that women who presented high CD45RA^+^/CD45RO^+^ and low CCL20^+^/CCR6^+^ ‘immunoscores’ would progress to CIN3 and CC. Indeed, this immunological profile together with clinical follow-up of HPV-infected women could enhance the real premalignant lesion diagnosis and may prevent the CC development. The correct diagnosis will certainly reduce an inappropriate surgical intervention, overtreatment, and psychological distress from unnecessary follow up. “Our study has a limitation. At IFF/Fiocruz, depending on CIN location and its grade, patients were released or followed for up to two years without other CIN development. It is considered persistence if the women have another cervical lesion in this 2-year interval, and recurrence, if she presents any cervical lesion after her clinical discharge. Thus, we have difficulty to determine the lesion progression, and consequently, we were not able to assess whether these ‘immunoscores’ may be used as prognostic markers. Therefore, we proponed a more comprehensive analysis of longitudinal studies that should be conducted to associate CD45RA^+^/CD45RO^+^ and CCL20^+^/CCR6^+^ ‘immunoscore’ to CIN progression and validate its value as prognostic methods.

## Materials

### Study population

From February 2012 to February 2016, eighty-one consecutive patients were enrolled for this study. Patients with suspicious of cervical premalignant lesion were seen in the colposcopy outpatient clinic of the Fernandes Figueira Woman, Child and Adolescent’s Health National Institute at Oswaldo Cruz Foundation, Rio de Janeiro, Brazil (IFF/Fiocruz). After histopathological analysis, patients were classified in CIN I, II and III and CC groups by certified pathologists.

All patients received free appropriate diagnosis and routine treatment and were followed up clinically to evaluate a possible CIN recurrence. Patients who had no cervical lesions in two years of follow-up were considered cured and released. The control group was composed of cervical biopsies from hysterectomized women, without histopathological HPV-associated lesions.

All volunteers provided written informed consent for participation in this study, approved by two Fiocruz Institutional Ethical Review-Boards (protocols number 14558313.4.0000.5262 and 14558313.8.3001.5269). All procedures performed in the studies were in accordance with the Helsinki declaration.

### DNA-HPV detection

Patients and control groups underwent a cervical cytobrush to obtain the genomic DNA. The DNA was isolated with the QIAGEN QIAamp DNA FFPE Tissue Kit (Qiagen, Valencia, CA) according to the manufacturer’s instructions. Briefly, each HPV PCR was performed in a 25 μl reaction mixture containing 50 ng of genomic DNA templates, 10 pmol/μl of each MY9-MY11 primer (Table 6), 2 mM of each deoxynucleoside triphosphate, 1X PCR buffer (50 mM KCl, 10 mM Tris–HCl, and 0.1% Triton X-100), 50 mM MgCl_2_, and 5 U/μl Taq polymerase (Promega Corporation, Madison, WI). The PCR profile consisted of an initial melting step at 95 °C for 5 min followed by 35 cycles at 94 °C for 1 min, 40 °C for 1 min, and 72 °C for 1 min, and a final extension step at 72 °C for 10 min. Human beta-globin was amplified in the same samples to control sample quality and adequacy. The PCR mix was performed in 25 μl reaction mixture containing 50 ng of genomic DNA templates, 10 pmol/μl of each primer GH20-PC04 (Table 2), 2 mM of each deoxynucleoside triphosphate, 10X PCR buffer, 50 mM MgCl_2_ and 5 U/μl Taq polymerase. The PCR profile consisted of an initial melting step at 95 °C for 5 min followed by 35 cycles at 94 °C for 1 min, at 60 °C for 1 min, and at 72 °C for 1 min, and a final extension step at 72 °C for 10 min. Both PCR products were submitted to electrophoresis in 1.5% agarose gel stained with ethidium bromide.

### Identification of CD45RA^+^, CD45RO^+^, CCR6^+^ and CCL20^+^ positive-cells in cervical lesion

Serial paraffin-embedded tissue sections (3 μm) were fixed in silane-coated slides (Sigma, Missouri, EUA). To determine the cell profile, immunoperoxidase staining was used according to the REVEAL Biotin-Free Polyvalent HRP manufacturer's instructions (Spring, CA, USA). Sections were incubated overnight at 4 °C with specific antibodies against human CD45RA^+^ (BD Biosciences, New Jersey, USA, clone HI100, dilution 1:10) and CD45RO^+^ (BD Biosciences, clone UCHL1, dilution 1:10), CCL20^+^ (Abcam, Cambridge, UK, clone EPR22376-58, dilution 1:10) and CCR6^+^ (Abcam, clone MM0066-3L1, dilution 1:15). Positive stained cells were counted in twenty fields (400 ×) in the intraepithelial (IE) area and in the marginal stroma (MS) area. For this study, we defined IE as the site of the epithelium, where it presents dysplastic areas, and MS the site of the stroma that borders the IE region evaluated. We limited analysis to the area directly surrounding the pathologist-identified dysplastic region and excluded areas with no dysplasia. Cell counts were performed using a grid (1 cm^2^ divided into 10 mm^2^) by two different observers.

### ‘Immunoscore’

**Figure 7:**
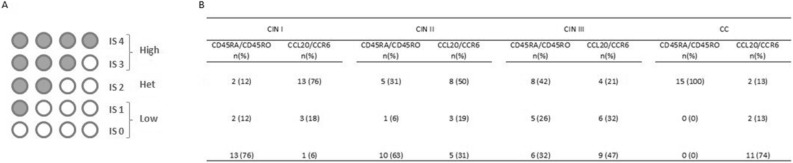
(**A**) ‘Immunoscore’ scheme classification. Using the median CD45RA^+^ or CD45RO^+^ cell density of all analyzed cervical lesions samples as cut-off value, samples were classified as high or low CD45RA^+^ and high or low CD45RO^+^ in IE and MS separately. Subsequently, samples were subdivided into five groups (0–IV) according to their CD45RA^+^ and CD45RO^+^ cell infiltration of IE and MS as described previously by Lechner et al.^51^. Gray colored dots represent scores for defined parameters in low (none or one higher parameter), het (two higher parameters), or high (three or four higher parameters) ‘immunoscores’. (**B**) Percentage of grouped patients according to lesion severity classified in the ‘immunoscore’ levels.

‘Immunoscore’ incorporated the number, type, and distribution of immune cells in cancer and CIN samples. Using these three factors, a score of I0 to I4 was given to the cervical lesions (Fig. 7). Herein, we classified a higher score according to number of studied cellular marker distribution (CD45RA^+^ and CD45RO^+^, CCL20^+^ and CCR6^+^, CD45RA^+^ and CCL20^+^, CD454RA^+^ and CCR6^+^, CD45RO^+^ and CCL20^+^, CD454RO^+^ and CCR6^+^) infiltration in both IE and MS.

To perform the ‘immunoscore’ in cervical lesions, we first determined the frequency of CD45RA^+^, CD45RO^+^, CCR6^+^ and CCL20^+^ expressing cells per mm^2^ in CIN and CC groups, in both cervical IE and MS areas. The median CD45RA^+^, CD45RO^+^, CCR6^+^ and CCL20^+^ cell densities of all analyzed samples in IE and MS were used as a cut-off value.

According to a combination between CD45RA^+^/CD45RO^+^ and CCL20^+^/CCR6^+^ analysis in IE and MS, independently, samples were classified as low (none or one higher parameter), het (two higher parameters) and high (three and four higher parameters—Fig. 7)^50^.

We used the χ^2^ test in a 3 × 2 contingency table to calculate statistical differences between the groups using GraphPad Prism (version 5).

### Statistical analysis

After examining the distribution of means in all of analyzed groups, nonparametric Kruskal–Wallis test and post-test of Dunns for multiple comparison were performed in GraphPad Prism 5.0 software (GraphPad) for comparisons between different continuous variables according to the categories in the study. It was considered statistically significant a *p* value < 0.05. We used the χ^2^ test, for a simple contingency table 2 × 2, to compare ordinary variables. Nonparametric Spearman test was used for correlation between CD45RA^+^ and CD45RO^+^ positive cells in epithelium and stroma marginal areas among groups. A correlation with *ρ* value from 0.7 to 1.0 was considered as strong association, 0.5–0.69 as a moderate and 0.3–0.49 as a weak association.

A multivariate analysis was performed to evaluate the influence of clinical and environmental data in the number of CD45RA^+^, CD45RO^+^, CCL20^+^ and CCR6^+^ expressing cells as well as CD45RA^+^/CD45RO^+^ and CCL20^+^/CCR6^+^ ‘immunoscores’. Multiple linear regression analysis was performed to determine the effect of clinical and environmental variables on the cell markers expression, and multiple logistic regression was applied to determine the influence of these variables among ‘immunoscore’ levels. Both multiple linear and logistic regression were performed considering age (in years), tobacco use, alcohol consumption and number of abortions as independent variables. These variables were chosen based on statistical differences observed in the analysis of the clinical and environmental data considering a *p* value < 0.20, as showed in Table 1. The analyses were performed in GraphPad Prism 9.0 software (GraphPad), and the effect of clinical variables were considered statistically significant when *p* value < 0.05.
